# Obstructive sleep apnea is associated with increased coronary plaque instability: an optical frequency domain imaging study

**DOI:** 10.1007/s00380-019-01363-8

**Published:** 2019-02-21

**Authors:** Takao Konishi, Yusuke Kashiwagi, Naohiro Funayama, Tadashi Yamamoto, Hironori Murakami, Daisuke Hotta, Shinya Tanaka

**Affiliations:** 1 Department of Cardiology, Hokkaido Cardiovascular Hospital, 1-30, West 13, South 27, Chuou-ku, Sapporo, 064-8622 Japan; 2 Department of Cancer Pathology, Hokkaido University Graduate School of Medicine, Sapporo, Japan; 3 Department of Cancer Pathology, Faculty of Medicine, Hokkaido University, Sapporo, Japan

**Keywords:** Obstructive sleep apnea, Plaque instability, Optical frequency domain imaging

## Abstract

Obstructive sleep apnea (OSA) is associated with coronary artery disease (CAD) and with an increased risk for myocardial infarction, stroke or death due to cardiovascular disease. Optical frequency-domain imaging (OFDI) is a useful modality for evaluating the characteristics of atherosclerotic plaque. The purpose of the study was to use OFDI to investigate the association of OSA with coronary plaque characteristics in patients undergoing percutaneous coronary intervention (PCI). We retrospectively analyzed OFDI data for coronary artery plaques from 15 patients with OSA and 35 non–OSA patients treated between October 2015 and October 2018. Plaque morphology was evaluated for 70 lesions, including 21 from patients with OSA and 49 from non–OSA patients. Compared with the non–OSA group, patients with OSA had significantly higher prevalences of thinned cap fibroatheroma (TCFA) (67% vs. 35%, *P* = 0.014) and microchannels (86% vs. 55%, *P* = 0.014); a significantly higher mean lipid index (1392 ± 982 vs. 817 ± 699, *P* = 0.021), macrophage grade (8.4 ± 6.4 vs. 4.8 ± 4.5, *P* = 0.030), and maximum number of microchannels (1.5 ± 1.0 vs. 0.7 ± 0.7, *P* = 0.001); and a significantly lower mean minimum fibrous cap thickness (69.4 ± 28.7 vs. 96.1 ± 51.8 μm, *P* = 0.008). This OFDI analysis suggests that OSA is associated with unstable plaque characteristics in patients with CAD. More intensive medical management for stabilization of coronary atherosclerotic plaque is required in patients with OSA.

**Electronic supplementary material:**

The online version of this article (10.1007/s00380-019-01363-8) contains supplementary material, which is available to authorized users.

## Introduction

Obstructive sleep apnea (OSA) is a common disorder with an estimated prevalence of 10–50% in men and 3–24% in women [1–4]. OSA is an independent risk factor for coronary artery disease (CAD), and up to 70% of patients with CAD have undiagnosed OSA [5]. Several studies have shown that OSA is associated with an increased risk for cardiovascular diseases such as myocardial infarction, stroke or death from cardiovascular disease [6–9].

Optical coherence tomography (OCT) and optical frequency domain imaging (OFDI) are intravascular imaging modalities that use reflection of near-infrared light to create images. These methods give images with high resolution of 10–20 µm, which is 10 times higher than that of intravascular ultrasound (IVUS). Recent OCT and OFDI studies have reported plaque rupture, lipid-rich plaques, thinned cap fibroatheroma (TCFA), cholesterol crystals, macrophage accumulation, and microchannels as characteristics of unstable plaque [4, 10–19].

Computed tomography (CT) in patients with OSA has revealed larger coronary plaque burdens and a larger lipid core compared to patients without OSA [20, 21]. IVUS has shown that patients with OSA have a larger coronary atherosclerotic plaque volume than patients without OSA [22]. OCT and OFDI are superior to these modalities due to their higher resolution in visualizing in-depth microstructure. In the current study, the association of OSA with features of plaque stability was examined using OFDI in patients undergoing percutaneous coronary intervention (PCI), as the first such study of this association.

## Methods

### Sample population

Data were analyzed retrospectively for 50 consecutive patients (37 men, 13 women) aged > 30 years who underwent OFDI-guided PCI at Hokkaido Cardiovascular Hospital between October 2015 and October 2018. For each participant, polysomnography (PSG) monitoring was performed within 6 months of OFDI-guided PCI. PSG monitoring was conducted in a standardized fashion. Overnight PSG was performed using an SAS-3200 (Nihon Kohden, Tokyo, Japan). A total of 21 lesions were examined in 15 patients with OSA based on an apnea hypopnea index (AHI) ≥ 15, and 49 lesions were examined in 35 patients without OSA (AHI < 15). The mean ages in the OSA and non–OSA groups were 71.8 ± 10.4 and 69.7 ± 8.9 years, respectively, with no significant difference between the groups. Patients presenting with left main CAD and cardiogenic shock were already excluded from 50 consecutive patients who underwent OFDI-guided PCI. Patients who accepted continuous positive airway pressure therapy (CPAP) as a treatment for OSA were also excluded. The study was approved by the Ethics Committee of Hokkaido Cardiovascular Hospital and was performed in compliance with the Declaration of Helsinki and ethical principles for medical research involving human subjects. All patients gave written informed consent.

## Coronary angiography

Coronary angiograms were analyzed by offline quantitative coronary angiography (GE ver. 5.10.1, Pie Medical Imaging BV. Maastricht, The Netherlands). Reference diameter, minimum lumen diameter, diameter stenosis, and lesion length were measured.

## OFDI method and analysis

An OFDI imaging catheter (FastView™, Terumo Corp., Tokyo, Japan) was advanced using a 0.014-inch guidewire with the help of a 6- or 7-Fr guiding catheter, and the imaging core was placed at a site distal to the lesion. Before PCI, OFDI was performed with continuous flush of contrast media at a rate of 4 mL/s, and the OFDI wire was pulled back at a rate of 20–40 mm/s. OFDI was generally performed without dilation by a balloon catheter, but the lesion was dilated with a small-sized balloon if the OFDI catheter could not pass through the lesion because of severe stenosis. For patients with acute coronary syndrome (ACS) without spontaneous recanalization, aspiration thrombectomy was performed prior to OFDI. The plaque morphology of culprit lesions was studied. Other lesions in the same epicardial artery with > 50% stenosis in angiography were also included in OFDI analysis. After identifying the most stenotic cross-section, the 5-mm proximal and distal cross-sections (total length: 10 mm) were examined. Cross-sectional images were then analyzed every 1 mm and evaluated for the presence of plaque rupture, plaque erosion, luminal thrombus, and categorized into lipid-rich plaque, TCFA, or a lesion with macrophage infiltration, cholesterol crystals, microchannels or calcification. OFDI analysis was conducted by two independent investigators (T.K. and R.K.) who were blinded to the clinical course of each patient. When there was discordance between the investigators, a consensus reading was obtained.

## Definitions of OFDI findings

**Fig. 1:**
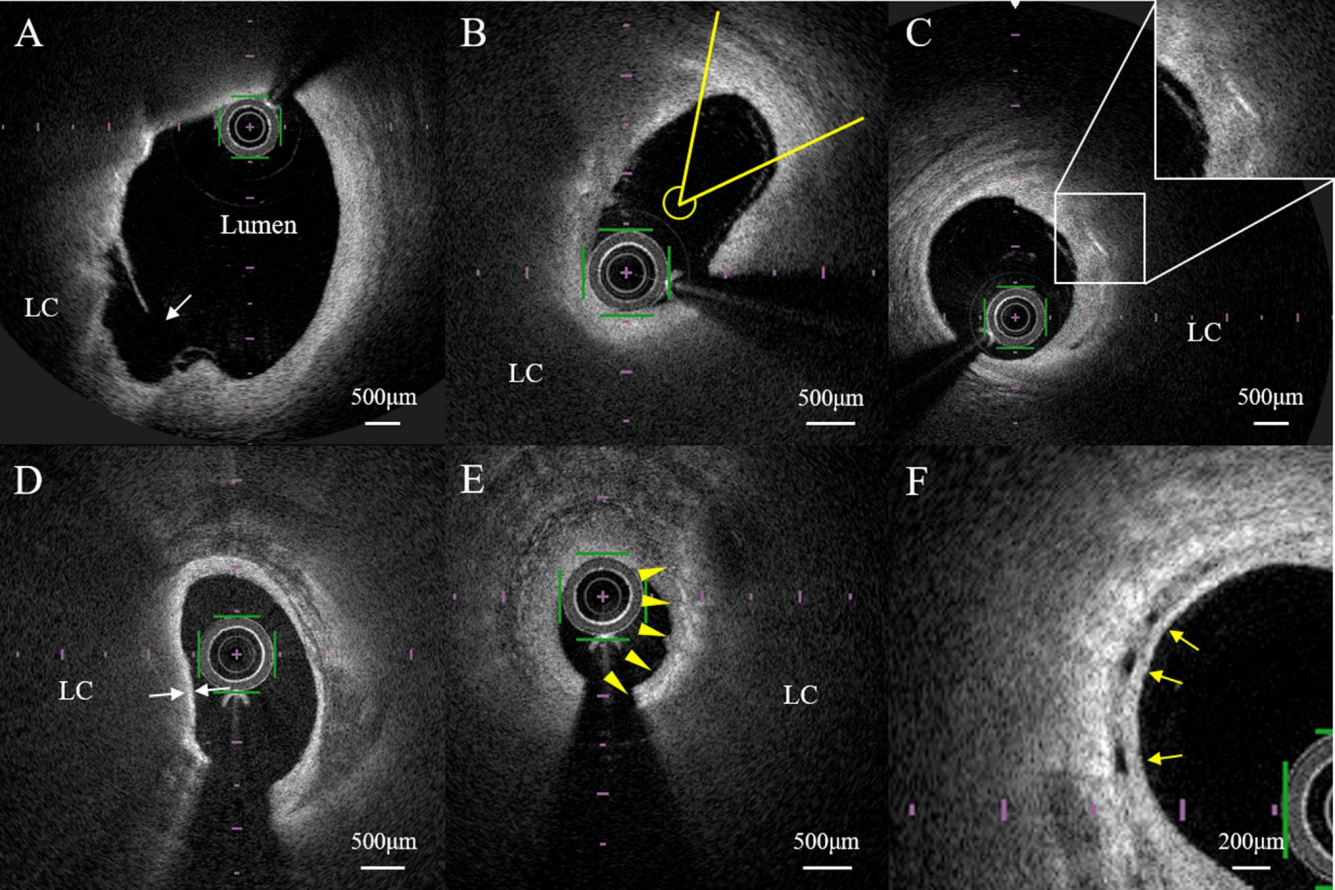
Representative plaque images from optical frequency domain imaging (OFDI).** a** Ruptured plaque (white arrow) with fibrous cap discontinuity leading to communication between the lipid core (LC) and lumen. **b** Measurement of the lipid arc for a lipid-rich plaque. The maximum lipid arc was measured within a lipid-rich plaque (yellow lines). **c** A cholesterol crystal was defined as a thin, linear region of high signal intensity within the lipid plaque, without backscattering. **d** Measurement of fibrous cap thickness. The thickness was measured at the thinnest point three times, and the average was taken (white arrows). **e** Macrophage accumulation was defined as increased signal intensity within the fibrous cap, accompanied by heterogeneous backward shadows (arrowheads). **f** Microchannels were defined as small vesicular or tubular structures with diameters of 50–300 μm within the intima

**Fig. 2:**
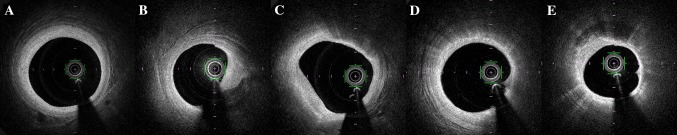
Semiquantification of macrophage accumulation in optical frequency domain imaging (OFDI). Representative cross–sectional OFDI images with the following grades: **a** grade 0, no macrophages; **b** grade 1, localized accumulation; **c** grade 2, clustered accumulation in < 1 quadrant; **d** grade 3, clustered accumulation in ≥ 1 and < 3 quadrants; and **e** grade 4, clustered accumulation in ≥ 3 quadrants

OFDI analysis revealed the presence of plaque rupture, plaque erosion, intraluminal thrombus, lipid-rich plaque, TCFA, macrophage accumulation, microvessels, and cholesterol crystals in the plaque. Plaque rupture was defined as the presence of fibrous cap discontinuity leading to communication between necrotic tissue and the lumen (Fig. 1a) [23, 24]. Plaque erosion was defined as a lesion without fibrous cap disruption and the presence of thrombus [25]. A thrombus was defined as a well-delineated mass with a high signal attached to the luminal surface or floating within the lumen [24]. Lipid-rich plaques were defined as lesions with a lipid arc > 180° (Fig. 1b) [24]. The lipid arc was measured within a lipid-rich plaque, and the maximum value was recorded (Fig. 1b). Lipid-core length was defined as the length of plaque with > 90° of lipid and was measured on the longitudinal view. The lipid index is the maximum lipid arc multiplied by the lipid-core length [26]. A cholesterol crystal was defined as a thin, linear region of high signal intensity within the lipid plaque, without backscattering (Fig. 1c) [24]. TCFA was defined as a fibrous cap thickness (FCT) < 65 μm, where FCT is the minimum thickness of a signal-rich layer from the coronary artery lumen to the inner border of the underlying lipid in the culprit lesion (Fig. 1d) [24]. Macrophage accumulation was defined as increased signal intensity within the fibrous cap, accompanied by heterogeneous backward shadows (Fig. 1e), and macrophages were semiquantified based on axial and circumferential distribution, as follows: grade 0, no macrophages; grade 1, localized macrophage accumulation; grade 2, clustered accumulation in < 1 quadrant; grade 3, clustered accumulation in ≥ 1 and < 3 quadrants; and grade 4, clustered accumulation in ≥ 3 quadrants (Fig. 2) [24, 27]. The range for the macrophage score was 0–40, based on summation of the 0–4 grades across all slices. Microchannels were defined as small vesicular or tubular structures with diameters of 50 to 300 μm within the intima (Fig. 1f) [24]. The number of microchannels was also counted at the cross-section with the highest number of microchannels [19]. Calcification was defined as well-delineated and low backscattered heterogeneous regions [24]. Spotty calcium deposits were defined as those with length < 4 mm and maximal arc < 90°, and deposits not meeting these criteria were classified as large calcium deposits [28, 29]. Microcalcification was defined as a small microcalcification with a maximal calcium angle < 22.5° and a maximal calcification length < 1 mm [30].

### Statistical analysis

Continuous variables are shown as means ± standard deviation (SD) or median (interquartile range) and categorical variables as counts and percentages. The normality of distributions was assessed by the Kolmogorov–Smirnov test. Between-group differences were examined by Pearson chi-square or Fisher exact test for categorical variables and Student *t* test or Mann–Whitney *U* test for continuous variables, as appropriate. *P* < 0.05 was considered to be significant. All data were analyzed with SPSS 25.0 (IBM Corp., Armonk, NY).

## Results

### Clinical characteristics

The clinical characteristics of the OSA and non–OSA groups are compared in Table 1. The mean AHI was significantly higher in the OSA group (30.9 ± 12.6 vs. 7.2 ± 3.8, *P* < 0.001). The mean LDL-C did not differ significantly between the groups (107 ± 29 vs. 104 ± 45 mg/dl, *P* = 0.732). All other characteristics, including medications and concomitant diseases, were similar in the two groups. All patients who had statin therapy prior to PCI were treated with strong statin (atorvastatin 10 mg/day, rosuvastatin 2.5–5 mg/day or pitavastatin 2 mg/day).

**Table 1:** Baseline characteristics in patients with and without obstructive sleep apnea

Item	Non-OSA (*n* = 35)	OSA (*n* = 15)	*P*-value
Age (years)	69.7 ± 8.9	71.8 ± 10.4	0.480
Male	27 (77)	10 (67)	0.493
Body mass index	23.6 ± 3.2	23.6 ± 3.3	0.986
Diabetes mellitus	11 (31)	4 (27)	0.736
Hypertension	23 (66)	12 (80)	0.502
Dyslipidemia	29 (83)	12 (80)	0.810
Chronic kidney disease	7 (20)	4 (27)	0.713
Current smoker	13 (37)	4 (27)	0.474
Apnea hypopnea index	7.2 ± 3.8	30.9 ± 12.6	< 0.001
Family history of coronary artery disease	4 (11)	3 (20)	0.415
History of PCI or CABG	15 (43)	5 (33)	0.529
History of myocardial infarction	11 (31)	1 (7)	0.079
History of TIA or cerebral infarction	3 (9)	0	0.545
History of peripheral artery disease	0	1 (7)	0.300
Prior statin use	11 (31)	6 (40)	0.558
Duration <3 months	0	0	–
3–12 months	3 (9)	2 (13)	0.607
≥12 months	8 (23)	4 (27)	0.942
Prior aspirin use	15 (43)	7 (47)	0.804
Prior clopidogrel use	11 (31)	2 (13)	0.294
Prior ACEI or ARB	9 (26)	6 (40)	0.333
Prior calcium channel blocker use	12 (34)	4 (27)	0.746
Prior beta blocker use	10 (29)	4 (27)	0.891
Prior eicosapentaenoic acid	4 (11)	1 (7)	0.607
Prior ezetimibe	4 (11)	0	0.302
Hemoglobin (g/dl)	13.5±1.5	13.3 ± 2.0	0.800
HbA1c (%)	6.4 ± 1.4	6.2 ± 1.1	0.662
Glucose (mg/dl)	140 ± 78	144 ± 64	0.882
LDL-C (mg/dl)	104 ± 45	107 ± 29	0.732
Triglyceride (mg/dl)	115 (86)	172 (136)	0.564
HDL-C (mg/dl)	49 ± 12	50 ± 14	0.936
LDL-C to HDL-C ratio	2.2 ± 1.1	2.3 ± 0.9	0.660
Acute coronary syndrome	18 (51)	10 (67)	0.320
ST-elevation myocardial infarction	9 (26)	6 (40)	0.333
Non ST-elevation myocardial infarction	9 (26)	4 (27)	0.944

Values are mean ± SD or number (%) of observations

*PCI* percutaneous coronary intervention, *CABG* coronary artery bypass grafting, *TIA* transient ischemic attack, *ACE*angiotensin-converting enzyme inhibitor, *ARB* angiotensin II receptor blocker

## Angiographic findings

Plaque location and angiographic data are shown for the two groups in Table 2. The OSA group had a significantly higher stenosis diameter (87.7% ± 14.6% vs. 79.7% ± 15.4%, *P* = 0.044). There were no significant differences in plaque location or in other angiographic data between the two groups.

**Table 2:** Angiographic characteristics in patients with and without obstructive sleep apnea

Item	Non-OSA (*n *= 49)	OSA (*n *= 21)	*P*-value
Plaque location			
Left anterior descending artery, *n* (%)	19 (39)	10 (48)	0.491
Left circumflex artery, *n* (%)	13 (27)	4 (19)	0.503
Right coronary artery, *n* (%)	17 (35)	7 (33)	0.912
Minimum lesion diameter, mm	1.21±0.57	0.92±0.59	0.060
Reference diameter, mm	2.90±0.57	2.93±0.42	0.789
Lesion length, mm	15.2±6.2	16.8±7.4	0.372
Diameter stenosis, %	79.7±15.4	87.7±14.6	0.044

## Plaque characteristics assessed by OFDI

**Fig. 3:**
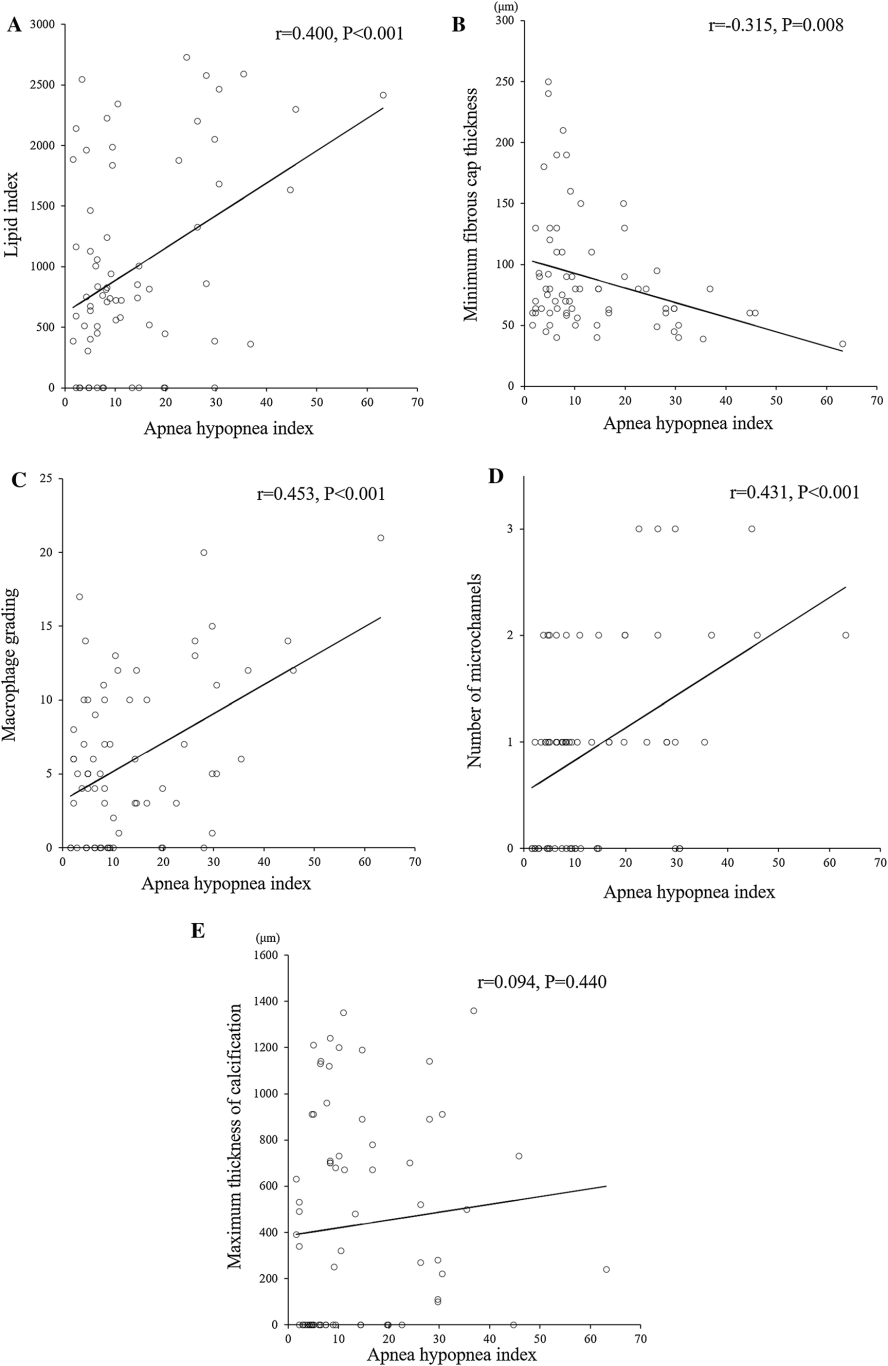
Relationships of apnea hypopnea index (AHI) with plaque characteristics. AHI was significantly correlated with **a** lipid index (*r* = 0.400, *P* < 0.001) **b** minimum fibrous cap thickness (*r* = − 0.315, *P* = 0.008) **c** macrophage grade (*r* = 0.453, *P* < 0.001), and **d** number of microchannels (*r* = 0.431, *P* < 0.001), but not with **e** maximum thickness of calcification (*r* = 0.094, *P* = 0.440)

The results of qualitative and semi-quantitative analysis of OFDI characteristics of the coronary plaques are shown in Table 3. Since the scores of macrophage grading and maximum number of microchannels were not distributed normally, Mann–Whitney *U*-test was used to compare differences between the two groups. Other continuous variables regarding OFDI characteristics were normally distributed. The OSA group had significantly higher prevalences of TCFA (67% vs. 35%, *P* = 0.014) and microchannels (86% vs. 55%, *P* = 0.014), a significantly higher mean lipid index (1392 ± 982 vs. 817 ± 699, *P* = 0.021), macrophage grade (8.4 ± 6.4 vs. 4.8 ± 4.5, *P* = 0.030), and maximum number of microchannels (1.5 ± 1.0 vs. 0.7 ± 0.7, *P* = 0.001), and a significantly lower mean minimum FCT (69.4 ± 28.7 vs. 96.1 ± 51.8 μm, *P* = 0.008) compared to the non–OSA group. AHI was positively correlated with the plaque characteristics of lipid index (*r* = 0.400, *P* < 0.001, Fig. 3a), macrophage grade (*r* = 0.453, *P* < 0.001, Fig. 3c), and number of microchannels (*r* = 0.431, *P* < 0.001, Fig. 3d), inversely correlated with the minimum FCT (*r* = − 0.315, *P* = 0.008; Fig. 3b), and not significantly correlated with the maximum thickness of calcification (*r* = 0.094, *P* = 0.440; Fig. 3e).

**Table 3:** OFDI characteristics in patients with and without obstructive sleep apnea

Item	Non-OSA (*n *= 49)	OSA (*n *= 21)	*P*-value
Number of subjects	35	15	
Number of plaques	49	21	
Plaques/subjects	1.4±0.5	1.4±0.6	0.872
Plaque rupture	6 (12)	4 (19)	0.473
Plaque erosion	6 (12)	3 (14)	0.815
Luminal thrombus	12 (24)	8 (38)	0.248
Lipid-rich plaque	18 (37)	10 (48)	0.394
Maximum lipid arc (degrees)	155 ± 71	184 ± 90	0.161
Lipid length (mm)	4.5 ± 3.1	6.3 ± 3.5	0.028
Lipid index	817 ± 699	1392 ± 982	0.021
Thin capped fibroatheroma (TCFA)	17 (35)	14 (67)	0.014
Minimum fibrous cap thickness (μm)	96.1±51.8	69.4±28.7	0.008
Macrophage infiltration	34 (69)	18 (86)	0.152
Macrophage grading	4.8±4.5	8.4±6.4	0.030
Cholesterol crystals	18 (37)	10 (48)	0.394
Microchannels	27 (55)	18 (86)	0.014
Maximum number of microchannels	0.7±0.7	1.5±1.0	0.001
Calcification	26 (53)	16 (76)	0.070
Large calcification	16 (33)	7 (33)	0.956
Spotty calcification	13 (27)	7 (33)	0.564
Maximum thickness of calcification	426±468	449±414	0.516
Microcalcification	5 (10)	6 (29)	0.075

## Risk factors for higher lipid index, TCFA, macrophage invasion, and microchannels

Multiple logistic regression analyses were performed to identify risk factors for higher lipid index, TCFA, macrophage invasion, and microchannels. Because history of PCI or CABG, history of myocardial infarction, prior aspirin use and prior clopidogrel use were correlated with prior statin use, they were excluded from the multiple variable analysis. We similarly excluded LDL-cholesterol (LDL-C) when we entered LDL-C to HDL-cholesterol (HDL-C) ratio in the multiple variable model. In multiple variable analyses, AHI, prior statin use, and glucose concentration were independently associated with a higher lipid index (Table 4); AHI and LDL-cholesterol (LDL-C) to HDL-cholesterol (HDL-C) ratio were associated with TCFA (Table 5); AHI and prior statin use were associated with greater macrophage grading (Table 6); and AHI, hemoglobin level and HDL-C were associated with greater microchannels (Table 7).

**Table 4:** Logistic regression analysis of lipid index

	Analysis			
	Single		Multiple	
	OR (95% CI)	*p*-value	OR (95% CI)	*p*-value
Age (years)	0.40 (0.13–1.22)	0.106		
Male	1.00 (0.34–2.92)	1.000		
Body mass index	2.67 (0.92–7.77)	0.072		
Diabetes mellitus	1.00 (0.35–2.82)	1.000		
Hypertension	1.93 (0.70–5.32)	0.206		
Dyslipidemia	0.56 (0.16–1.93)	0.360		
Chronic kidney disease	1.93 (0.62–6.07)	0.259		
Current smoker	1.30 (0.47–3.59)	0.607		
Obstructive sleep apnea	2.67 (0.92–7.77)	0.072		
Apnea hypopnea index	6.30 (1.61–24.7)	0.008	8.08 (1.70–38.5)	0.009
Family history of coronary artery disease	2.67 (0.63–11.3)	0.183		
History of PCI or CABG	0.43 (0.16–1.15)	0.092		
History of myocardial infarction	0.25 (0.08–0.80)	0.020		
Prior statin use	0.20 (0.07–0.59)	0.004	0.18 (0.05–0.62)	0.007
Prior aspirin use	0.44 (0.17–1.16)	0.096		
Prior clopidogrel use	0.35 (0.12–1.07)	0.065		
Prior ACEI or ARB	0.42 (0.14–1.24)	0.117		
Prior calcium channel blocker use	0.66 (0.24–1.86)	0.435		
Prior beta blocker use	0.77 (0.28–2.11)	0.607		
Prior eicosapentaenoic acid	0.36 (0.07–2.02)	0.247		
Prior ezetimibe	0.23 (0.02–2.15)	0.197		
Hemoglobin (g/dl)	2.36 (0.81–6.93)	0.117		
HbA1c (%)	2.67 (0.63–11.3)	0.183		
Glucose (mg/dl)	3.63 (1.20–10.9)	0.022	6.22 (1.62–23.8)	0.008
LDL-C (mg/dl)	2.01 (0.77–5.22)	0.152		
Triglyceride (mg/dl)	1.83 (0.61–5.47)	0.277		
HDL-C (mg/dl)	0.60 (0.19–1.91)	0.385		
LDL-C to HDL-C ratio	2.67 (0.92–7.77)	0.072		
Acute coronary syndrome	2.65 (0.99–7.11)	0.054		
ST-elevation myocardial infarction	2.18 (0.79–5.96)	0.134		
Non ST-elevation myocardial infarction	1.35 (0.46–3.96)	0.585		

*OR* odds ratio, *CI* confidence interval, *PCI* percutaneous coronary intervention, *CABG* coronary artery bypass grafting, *TIA* transient ischemic attack, *ACEI* angiotensin-converting enzyme inhibitor, *ARB* angiotensin II receptor blocker

**Table 5:** Logistic regression analysis of TCFA

	Analysis			
	Single		Multiple	
	OR (95% CI)	*p*-value	OR (95% CI)	*p*-value
Age (years)	2.21 (0.83–5.89)	0.113		
Male	1.35 (0.45–4.02)	0.593		
Body mass index	0.51 (0.20–1.34)	0.174		
Diabetes mellitus	0.43 (0.14–1.29)	0.133		
Hypertension	1.37 (0.50–3.78)	0.544		
Dyslipidemia	0.62 (0.19–2.09)	0.444		
Chronic kidney disease	1.87 (0.61–5.77)	0.276		
Current smoker	1.07 (0.39–2.96)	0.894		
Obstructive sleep apnea	3.77 (1.28–11.1)	0.016		
Apnea hypopnea index	11.7 (2.37–57.6)	0.003	7.55 (1.35–42.4)	0.022
Family history of coronary artery disease	0.49 (0.12–2.08)	0.333		
History of PCI or CABG	0.39 (0.14–1.05)	0.063		
History of myocardial infarction	0.48 (0.16–1.46)	0.196		
Prior statin use	0.20 (0.06–0.64)	0.006	0.46 (0.12–1.68)	0.238
Prior aspirin use	0.87 (0.34–2.23)	0.767		
Prior clopidogrel use	0.66 (0.22–1.94)	0.446		
Prior ACEI or ARB	0.78 (0.27–2.24)	0.648		
Prior calcium channel blocker use	0.92 (0.33–2.58)	0.875		
Prior beta blocker use	0.82 (0.29–2.27)	0.700		
Prior eicosapentaenoic acid	0.47 (0.09–2.60)	0.386		
Prior ezetimibe	0.29 (0.03–2.75)	0.282		
Hemoglobin (g/dl)	0.39 (0.10–1.49)	0.169		
HbA1c (%)	0.39 (0.14–1.05)	0.063		
Glucose (mg/dl)	0.40 (0.13–1.19)	0.100		
LDL-C (mg/dl)	3.02 (1.09–8.40)	0.033		
Triglyceride (mg/dl)	0.38 (0.14–1.01)	0.053		
HDL-C (mg/dl)	0.38 (0.14–1.01)	0.053		
LDL-C to HDL-C ratio	7.11 (2.09–24.2)	0.002	4.53 (1.15–17.8)	0.030
Acute coronary syndrome	1.41 (0.53–3.71)	0.492		
ST-elevation myocardial infarction	3.13 (1.12–8.71)	0.029	1.97 (0.57–6.77)	0.284
Non ST-elevation myocardial infarction	0.39 (0.12–1.23)	0.108		

*OR* odds ratio, *CI* confidence interval, *PCI* percutaneous coronary intervention, *CABG* coronary artery bypass grafting, *TIA* transient ischemic attack, *ACEI* angiotensin-converting enzyme inhibitor, *ARB* angiotensin II receptor blocker

**Table 6:** Logistic regression analysis of macrophage infiltration

	Analysis			
	Single		Multiple	
	OR (95% CI)	*p*-value	OR (95% CI)	*p*-value
Age (years)	3.23 (0.93–11.3)	0.066		
Male	1.26 (0.43–3.69)	0.672		
Body mass index	0.48 (0.15–1.53)	0.215		
Diabetes mellitus	1.04 (0.37–2.95)	0.940		
Hypertension	1.14 (0.42–3.09)	0.804		
Dyslipidemia	0.46 (0.13–1.67)	0.237		
Chronic kidney disease	1.11 (0.36–3.41)	0.858		
Current smoker	1.75 (0.62–4.94)	0.290		
Obstructive sleep apnea	2.08 (0.72–6.05)	0.177		
Apnea hypopnea index	7.80(1.61–37.9)	0.011	5.97 (1.10–32.5)	0.039
Family history of coronary artery disease	2.18 (0.52–9.25)	0.289		
History of PCI or CABG	0.32 (0.12–0.85)	0.023		
History of myocardial infarction	0.38 (0.13–1.12)	0.078		
Prior statin use	0.20 (0.07–0.58)	0.003	0.23 (0.07–0.73)	0.012
Prior aspirin use	0.19 (0.07–0.52)	0.001		
Prior clopidogrel use	0.27 (0.09–0.84)	0.024		
Prior ACEI or ARB	0.79 (0.28–2.22)	0.649		
Prior calcium channel blocker use	0.68 (0.24–1.90)	0.465		
Prior beta blocker use	0.45 (0.16–1.27)	0.132		
Prior eicosapentaenoic acid	0.60 (0.12–2.91)	0.526		
Prior ezetimibe	1.29 (0.20–8.21)	0.791		
Hemoglobin (g/dl)	2.25 (0.78–6.49)	0.133		
HbA1c (%)	3.39 (0.65–17.6)	0.147		
Glucose (mg/dl)	3.00 (1.08–8.34)	0.035	2.95 (0.93–9.39)	0.066
LDL-C (mg/dl)	2.32 (0.77–6.95)	0.133		
Triglyceride (mg/dl)	0.41 (0.15–1.08)	0.071		
HDL-C (mg/dl)	0.60 (0.22–1.64)	0.317		
LDL-C to HDL-C ratio	1.93 (0.69–5.44)	0.212		
Acute coronary syndrome	1.33 (0.51–3.48)	0.557		
ST-elevation myocardial infarction	0.77 (0.29–2.07)	0.603		
Non ST-elevation myocardial infarction	2.00 (0.65–6.13)	0.225		

*OR* odds ratio, *CI* confidence interval, *PCI* percutaneous coronary intervention, *CABG* coronary artery bypass grafting, *TIA* transient ischemic attack, *ACEI* angiotensin-converting enzyme inhibitor, *ARB* angiotensin II receptor blocker

**Table 7:** Logistic regression analysis of microchannels

	Analysis			
	Single		Multiple	
	OR (95% CI)	*p*-value	OR (95% CI)	*p*-value
Age (years)	4.19 (0.50–35.1	0.187		
Male	0.26 (0.08–0.85)	0.025	0.33 (0.06–1.69)	0.182
Body mass index	2.13 (0.61–7.43)	0.234		
Diabetes mellitus	1.52 (0.47–4.88)	0.482		
Hypertension	1.23 (0.38–4.05)	0.728		
Dyslipidemia	0.67 (0.18–2.52)	0.547		
Chronic kidney disease	0.66 (0.16–2.66)	0.558		
Current smoker	0.38 (0.10–1.51)	0.170		
Obstructive sleep apnea	5.46 (1.69–17.6)	0.005		
Apnea hypopnea index	8.04 (2.36–27.3)	0.001	9.44 (1.99–44.9)	0.005
Family history of coronary artery disease	2.41 (0.59–9.84)	0.220		
History of PCI or CABG	0.99 (0.33–2.99)	0.981		
History of myocardial infarction	0.50 (0.13–1.97)	0.318		
Prior statin use	1.48 (0.48–4.57)	0.493		
Prior aspirin use	0.53 (0.17–1.63)	0.525		
Prior clopidogrel use	0.28 (0.06–1.38)	0.118		
Prior ACEI or ARB	1.06 (0.32–3.51)	0.930		
Prior calcium channel blocker use	0.42 (0.11–1.64)	0.211		
Prior beta blocker use	0.38 (0.10–1.51)	0.170		
Prior eicosapentaenoic acid	1.28 (0.23–7.29)	0.781		
Prior ezetimibe	0.77 (0.08–7.36)	0.817		
Hemoglobin (g/dl)	0.12 (0.04–0.42)	0.001	0.18 (0.04–0.79)	0.022
HbA1c (%)	1.86 (0.62–5.59)	0.271		
Glucose (mg/dl)	6.32 (0.77–51.9)	0.086		
LDL-C (mg/dl)	1.88 (0.62–5.73)	0.266		
Triglyceride (mg/dl)	0.58 (0.19–1.75)	0.334		
HDL-C (mg/dl)	6.72 (1.77–25.5)	0.005	5.31 (1.03–27.3)	0.046
LDL-C to HDL-C ratio	0.33 (0.09–1.13)	0.078		
Acute coronary syndrome	0.68 (0.23–2.05)	0.496		
ST-elevation myocardial infarction	0.33 (0.08–1.28)	0.108		
Non ST-elevation myocardial infarction	1.86 (0.57–6.09)	0.303		

*OR* odds ratio, *CI* confidence interval, *PCI* percutaneous coronary intervention, *CABG* coronary artery bypass grafting, *TIA* transient ischemic attack, *ACEI* angiotensin-converting enzyme inhibitor, *ARB* angiotensin II receptor blocker

## Correlations among microchannels, macrophage grade, and FCT

There were significant correlations among microchannels, macrophages and FCT. Macrophage grading was positively correlated with the number of microchannels (*r* = 0.383, *P* = 0.001; Supplemental Fig. 1), and FCT was inversely correlated with the macrophage grade (*r* = − 0.415, *P* < 0.001; Supplemental Fig. 2).

## Observer variabilities

OFDI images were analyzed by two independent observers. The inter-observer reliabilities and intra-observer reproducibilities measured by the Pearson coefficient were *r* = 0.90 and 0.91 for lipid index, *r* = 0.92 and 0.94 for minimum FCT, *r* = 0.95 and 0.93 for macrophage grading, and *r* = 0.92 and 0.95 for maximum number of microchannels, respectively.

## Discussion

The main findings in this study were (1) that patients with OSA had a larger lipid burden, thinner fibrous cap, greater macrophage accumulation, and more microchannels compared to those without OSA; (2) lipid index, minimum FCT, macrophage accumulation and microchannels were positively or inversely correlated with AHI; and (3) in patients undergoing PCI, AHI, prior statin use and glucose concentration were predictors of lipid index; AHI and LDL-C to HDL-C ratio were predictors of TCFA; AHI and prior statin use were predictors of macrophage grading; and AHI, hemoglobin and HDL-C were predictors of greater microchannels. To the best of our knowledge, this study is the first in depth comparison of coronary artery plaques in patients with and without OSA, with analysis of correlations of AHI with characteristics of unstable plaque using OFDI in patients who underwent PCI. These observations improve understanding of the pathophysiology of OSA, and may have important implications for management of patients with OSA presenting with CAD.

## Comparison with previous studies

Our results are concordant with OFDI data from previous studies, with microchannels, macrophage accumulation, and TCFA found in 37–60%, 30–74%, and 11–34% of patients who underwent PCI, respectively [31–33]. FCT measured by OCT was significantly lower in plaques with positive remodeling and in low-attenuation plaques on CT angiography, compared with two-feature–negative plaques (76 ± 24 vs. 192 ± 49 μm, *P* < 0.001) [34].

## Lipid-rich plaque

A large lipid core is an important contributor to plaque rupture through mechanically increasing the tension of the fibrous cap covering the lipid core, leading to disruption [35]. In patients with OSA, intermittent hypoxia (IH) during sleep can increase oxidative stress, leading to oxidative modification of lipoproteins and other molecules [36–38]. These oxidized particles cause endothelial surface injury and promote accumulation of cholesterol in atherosclerotic plaque [39, 40]. CT studies have shown larger coronary plaque burdens and a larger lipid core in non–culprit lesions of patients with OSA compared to those in patients without OSA [20, 21], which is consistent with our data (Table 3, Fig. 3A).

## TCFA and FCT

Previous reports have shown that a thin fibrous cap is one of the most important features of unstable plaque in coronary and carotid artery [41–43]. Since matrix metalloproteinases (MMPs) released by macrophages induce thinning of fibrous caps of atherosclerotic plaques through collagen breakdown [44], more macrophage accumulation in the fibrous cap might lead to a thinner fibrous cap (Supplemental Fig. 2). Therefore, more macrophage accumulation in the plaque might contribute to a higher prevalence of TCFA and a lower FCT in patients with OSA (Table 3, Fig. 3b).

## Macrophage accumulation

In a murine model of OSA, IH during sleep caused recruitment of more macrophages to the aortic wall [45, 46], and IH during sleep for 6 weeks led to a significant increase in the percentage of macrophages in the aortic wall (6.4% ± 0.3% vs. 8.1% ± 0.3%, *P* = 0.003) [46]. Macrophages are thought to be extravasated from intraplaque microvessels into plaque tissue [47]. The number of microchannels in the plaque was weakly, but positively, correlated with the macrophage grade (Supplemental Fig. 1). Therefore, more intraplaque microvessels and direct invasion from the lumen of coronary arteries might contribute to greater macrophage accumulation in patients with OSA (Table 3, Fig. 3c).

## Microchannels

Serum concentrations of vascular endothelial growth factor (VEGF) and endothelin-1, which have important roles in angiogenesis, are increased in patients with OSA [48, 49]. Oxidative stress due to hypoxia in these patients induces endothelial dysfunction, leading to endothelial-derived microparticle (MP) formation [50]. MPs are small plasma membrane vesicles with diameters of about 0.05–1.00 μm that are released by plasma membranes of cells such as leukocytes, platelets, endothelial cells, erythrocytes and smooth muscle cells in response to damage [51]. Ayers et al. showed that the concentrations of platelet- and leukocyte-derived MPs were elevated in patients with OSA [52], and Tual-Chalot et al. found that MPs from patients with OSA induced an increase of angiogenesis through VEGF- and endothelin-1-mediated pathways [49]. These mechanisms might explain the finding of more microchannels in patients with OSA and CAD (Table 3, Fig. 3d).

## Unstable plaque in OSA and clinical perspectives

Several studies have shown that OSA might cause or accelerate atherosclerosis [53, 54]. In this study, multiple regression analysis showed that AHI is independently correlated with features of unstable plaque (Table 4, 5, 6, 7). These results support the hypothesis that OSA plays an important role in development of atherosclerotic plaque and plaque instability. However, in the Sleep Apnea Cardiovascular Endpoints (SAVE) trial, CPAP did not result in a lower incidence of the primary end point than usual care alone [55]. In this trial, the mean duration of CPAP use was only 3.3 h per night, which might not be sufficient for preventing cardiovascular events. After propensity-score matching, the incidence of a cerebrovascular event was significantly lower in 561 patients who used CPAP for more than 4 h per night, compared to that in the usual-care group (hazard ratio, 0.52, *P* = 0.02) [55]. Furthermore, there is evidence that treatment of OSA with CPAP improves endothelial function and reduces the risk for cardiovascular events [56], and a randomized controlled trial showed that CPAP therapy resulted in a significant reduction of carotid intima-media thickness [57]. Therefore, CPAP therapy and stringent management of other coronary risk factors might be effective for stabilization of unstable plaque and secondary prevention of adverse cardiovascular events in patients with OSA.

## Limitations

The sample size of this retrospective, cross–sectional study conducted at a single medical center was small, and the results require confirmation in a prospective study including a larger number of patients. Second, we could not analyze microchannels of < 50 μm due to the limit of the device resolution. Third, there is an inherent discrepancy between characteristics assessed by OFDI and actual histopathological findings [58]. Further analyses using higher resolution OFDI might enable more detailed assessment of intraplaque microstructures in patients undergoing PCI. Fourth, we did not have information on inflammatory biomarkers such as high–sensitivity CRP, ICAM-1 and E-selectin. Fifth, the analysis of the risk factors was done on a per lesion basis although the patient characteristics were analyzed on a per patient basis.

## Conclusion

This OFDI analysis suggests that OSA is associated with plaque instability in patients with CAD. More intensive medical management is required for patients with OSA for stabilization of coronary atherosclerotic plaques.

## Electronic supplementary material

Below is the link to the electronic supplementary material.

Supplementary material 1 (TIF 89 kb)

Supplementary material 2 (TIF 106 kb)
