# Supplementary figures and images for: Obstructive sleep apnea is associated with increased coronary plaque instability: an optical frequency domain imaging study
Source: Heart Vessels. 2019 Feb 21;34(8):1266–79. doi: 10.1007/s00380-019-01363-8 (PMC6620247; doi:10.1007/s00380-019-01363-8)

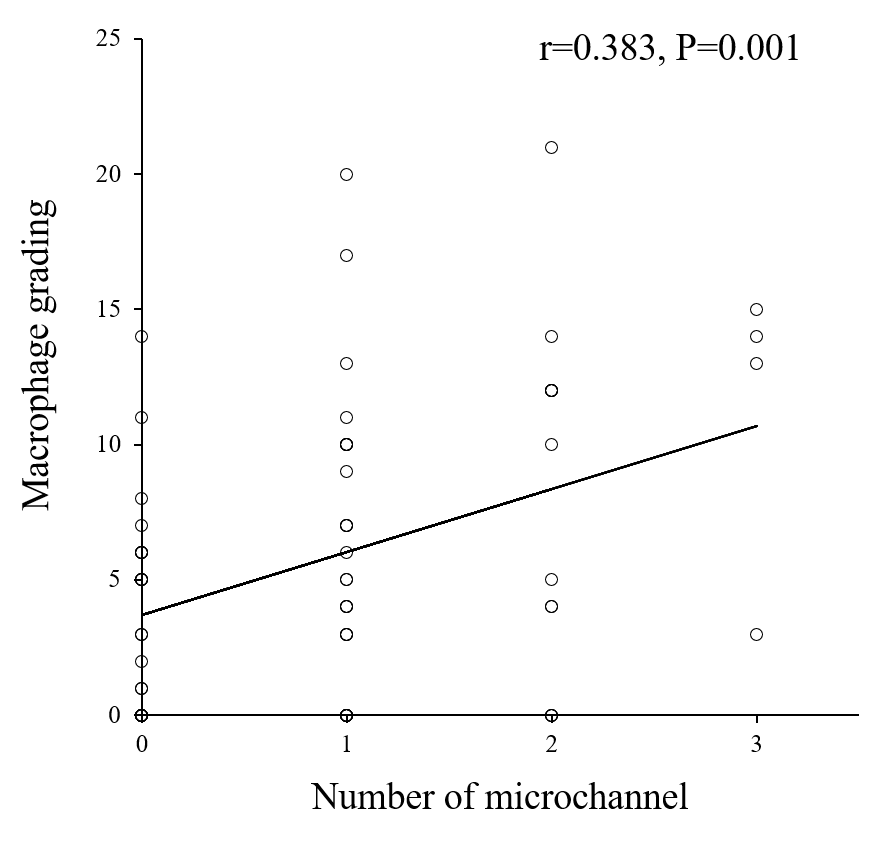

Supplement: Supplementary file 1 — Supplementary material 1 (TIF 89 kb) [file 380_2019_1363_MOESM1_ESM.tif]

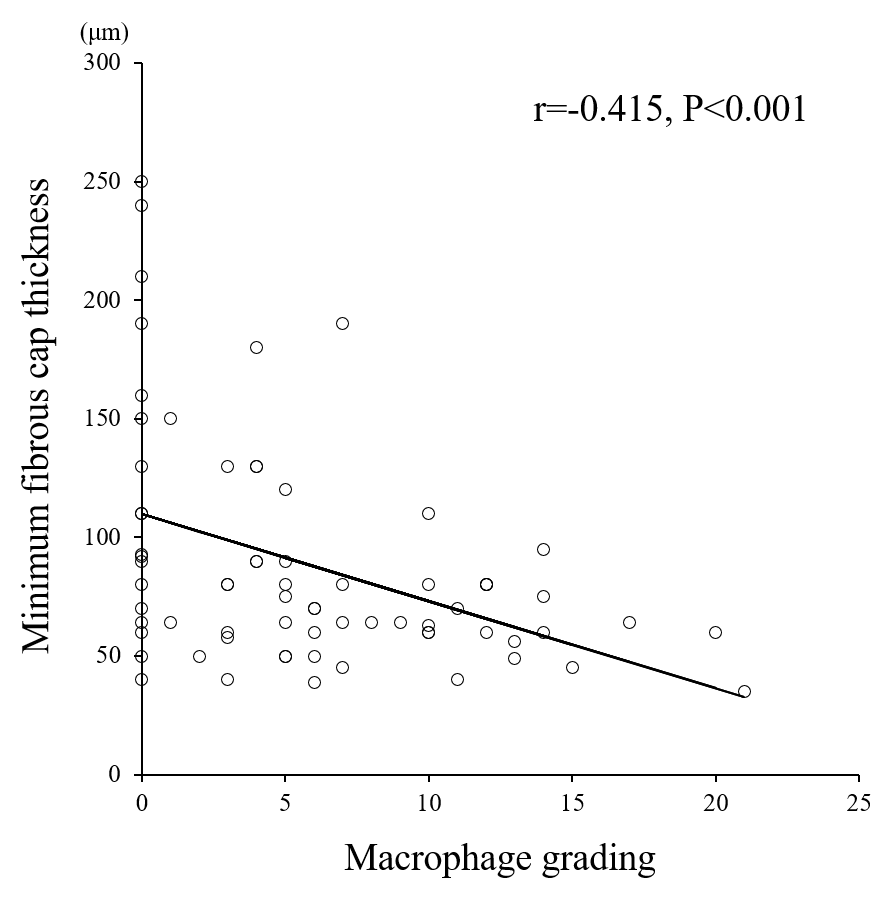

Supplement: Supplementary file 2 — Supplementary material 2 (TIF 106 kb) [file 380_2019_1363_MOESM2_ESM.tif]
